# Simulation of Phosphorus Chemistry, Uptake and Utilisation by Winter Wheat

**DOI:** 10.3390/plants8100404

**Published:** 2019-10-09

**Authors:** Lianhai Wu, Martin Blackwell, Sarah Dunham, Javier Hernández-Allica, Steve P. McGrath

**Affiliations:** 1 Sustainable Agriculture Sciences, Rothamsted Research, Okehampton EX20 2SB, UK; martin.blackwell@rothamsted.ac.uk; 2 Sustainable Agriculture Sciences, Rothamsted Research, Harpenden AL5 2JQ, UK; sarah.dunham@rothamsted.ac.uk (S.D.); javier.hernandez@rothamsted.ac.uk (J.H.-A.); steve.mcgrath@rothamsted.ac.uk (S.P.M.)

**Keywords:** phosphorus cycling, SPACSYS, modelling, winter wheat

## Abstract

The phosphorus (P) supply from soils is crucial to crop production. Given the complexity involved in P-cycling, a model that can simulate the major P-cycling processes and link with other nutrients and environmental factors, e.g., soil temperature and moisture, would be a useful tool. The aim of this study was to describe a process-based P module added to the SPACSYS (Soil Plant and Atmosphere Continuum System) model and to evaluate its predictive capability on the dynamics of P content in crops and the impact of soil P status on crop growth. A P-cycling module was developed and linked to other modules included in the SPACSYS model. We used a winter wheat (*Triticum aestivum, cv* Xi-19) field experiment at Rothamsted Research in Harpenden to calibrate and validate the model. Model performance statistics show that the model simulated aboveground dry matter, P accumulation and soil moisture dynamics reasonably well. Simulated dynamics of soil nitrate and ammonium were close to the observed data when P fertiliser was applied. However, there are large discrepancies in fields without P fertiliser. This study demonstrated that the SPACSYS model was able to investigate the interactions between carbon, nitrogen, P and water in a single process-based model after the tested P module was implemented.

## 1. Introduction

Phosphorus (P) supply from soils is crucial to crop production. Often, crop yields are constrained if no external P is supplied. The limitation of P on biomass production will become more severe under increasing nitrogen (N) global loading in the future [1]. Phosphorus fertiliser is mainly produced from rock phosphate, which is not only a finite resource, but also a geographically unevenly distributed one, meaning that continued supply is subject to geopolitical threats [2]. To support the demand for food with the projected increase in population this century [3], an increase in P fertiliser application will be expected [4]. Both limited supply and increased demand implies the importance of using the existing resources wisely in order to make agricultural production sustainable. The recovery of added P by crops is often low [5,6], due to sorption reactions of P which form poorly soluble or insoluble compounds [7]. Hence, there is a challenge in agriculture on how crop P use efficiency can be improved. P fertilisers can also contribute to P surpluses in soils and to P exports from land to surface waters. Therefore, an efficient way of maintaining optimum crop production through adding P fertilisers while reducing P losses to the environment is to manage P applications properly to ensure maximum crop acquisition of soil and fertiliser P [8].

Total P in soils consists of many different fractions, including crystalline, occluded and adsorbed particulate organic, soluble organic and soluble inorganic P. Soil organic P compounds have been largely overlooked in P agronomy, but can represent up to 90% of soil total P [9]. Methods have been developed to characterise soil organic P mainly focused on the identification and quantification of inositol phosphates [10] and other phosphomonoesters [11]. However, a small proportion of soil organic P compounds with potentially very different properties remain unidentified [11,12]. For the identified compounds, their cycling, mobility and bioavailability generally are poorly understood [10]. On the other hand, inorganic P exists in soils in various forms that are categorised in terms of accessibility (accessible to plants) and extractability (extractable in different reagents) in agricultural soils [13]. Furthermore, mobility, stability and transformations between P compounds are affected by soil environmental conditions, e.g., soil acidity [14] and land use [15]. Soil P dynamics are also influenced by complex interactions between physical, chemical and biological processes that occur within the rhizosphere. 

Given the complexity of P-cycling, a model that is able to simulate the major P-cycling processes and link with other nutrients and factors, would be a useful tool to quantify the impact of nutrients, soil moisture, field management practices and climatic conditions on crop growth and yields. There are several field-scale and process-based P-cycling models which are either independent ICECREAM [16,17] or modules integrated into other nutrient cycling models, e.g., ANIMO [18], APSIM [19], EPIC [20], CENTURY [21] and its daily version DAYCENT [22], *ecosys* [23] and other unnamed models [24,25]. Daroub et al. [26] also developed a soil P module that operates with two comprehensive crop simulation models within the DSSAT (Decision Support System for Agrotechnology Transfer) software [27]. Lewis and McGechan [28] reviewed some of these models in terms of the concepts and constituent processes and concluded that a comprehensive description of all processes relevant to P in soil should consider transport of both soluble and particulate P, inorganic and organic P, as well as transformations from one form of P to another following applications of both mineral fertiliser and manure P. However, they also concluded that a mechanistic approach is too complex for incorporation into a systems model for the whole range of P processes. 

SPACSYS [29,30] is a field scale model with a flexible soil layer definition (layer number and thickness of each defined layer), weather-driven, process-based and daily-time-step dynamic simulation on plant growth and development, soil carbon (C) and N cycling, with water movement and heat transfer. It has been used to investigate various issues, including nitrate leaching [31,32], root systems [33], greenhouse gas emissions [30,34], soil C and N stock change [35] and the responses of cropping/grassland systems to environmental changes [36,37]. However, it would be impossible to accurately assess the impacts of general management practices (i.e., combined application of N and P fertilisers) on agricultural systems using the SPACSYS model without incorporating P-cycling. The objectives of this study were to describe a process-based P module added to the SPACSYS model and to evaluate its predictive capability on the dynamics of P content in crops and the impact of soil P status on crop growth via validation with a winter wheat field experiment conducted at Rothamsted Research, Harpenden, UK, over two consecutive growing seasons between 2012 and 2014.

## 2. Results

**Figure 1:**
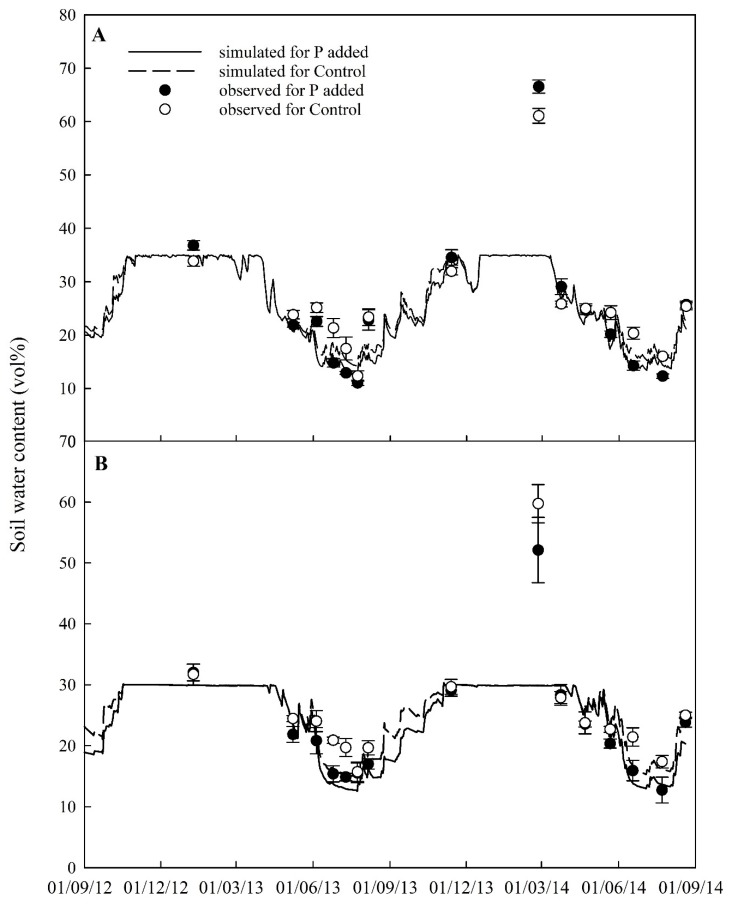
Comparison of soil water contents in the top (**A**: 0–23 cm) and the subsoil (**B**: 23–46 cm) between simulation and observation (three replications) covering two growing seasons for both Control and P added treatments. The error bars represent the standard deviation.

Accurately estimating the dynamics of soil moisture is essential for quantifying crop growth and nutrient cycling correctly. Simulated soil water content in separate soil layers over the growing seasons were compared with discrete samplings (Figure 1). Both simulated results and observed data show that soil moisture in the field of the Control treatment was higher than that of the P-added treatment, especially in the sub-soil layer (23–46 cm). The model performance statistical analysis demonstrate that the simulations followed the observed trend and reasonably matched the observed data (Table 1). It was noted that there were abnormal observed values (over 60% in the topsoil layer and between 45–63% in the subsoil layer on 25 February 2014). The reason is that soil was too wet to be sieved. Removal of the point, statistical analysis showed significantly improved model performance (Table 1). 

**Figure 2:**
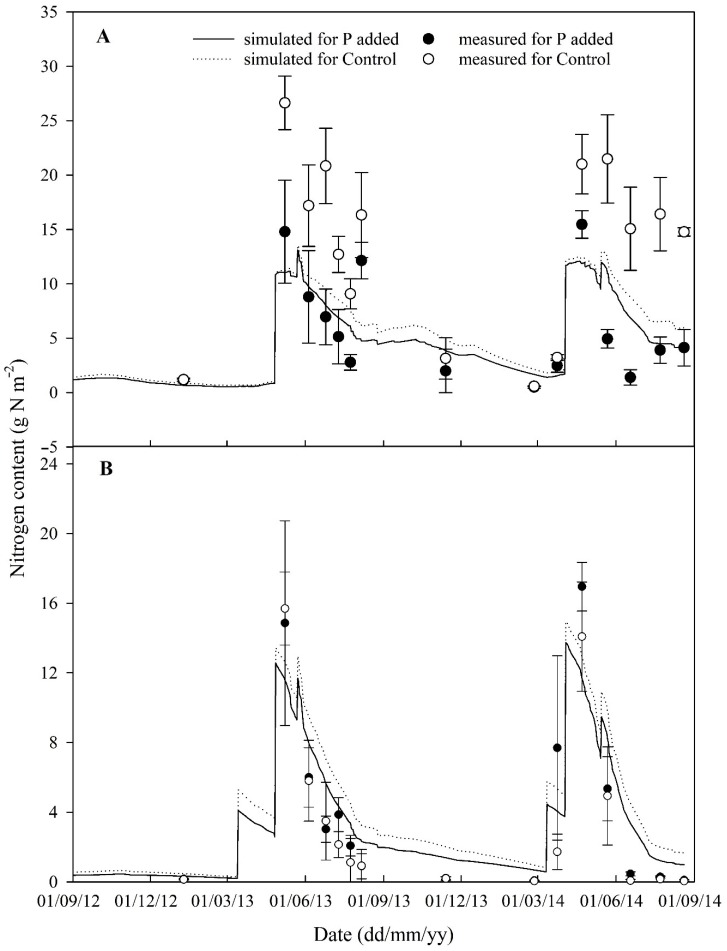
Comparison of nitrate (**A**) and ammonium (**B**) contents in the top soil (0–23 cm) between simulation and observation (three replications) over the two growing seasons for both Control and P added treatments. Error bars represent the standard deviation.

Similarly to soil water content, both simulated and measured nitrate and ammonium content in the topsoil showed the same trend through the growing seasons. In general, the contents from the P added treatment were lower than those from the control, especially for nitrate (Figure 2). The measured nitrate content in the Control treatment was much higher than that in the P-added treatment. However, the simulations did not show this. In general, the simulation of nitrate content for the P added treatment was over-estimated but it was under-estimated for the Control treatment.

**Figure 3:**
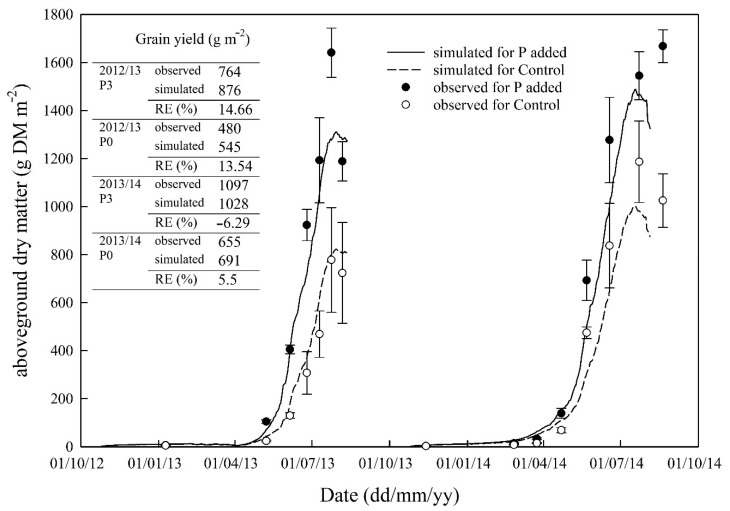
Simulated and observed (three replications) aboveground dry matter accumulation and leaf weight over two growing seasons. The error bars represent the standard deviation.

Overall, dynamics of aboveground dry matter for both treatments were simulated reasonably well compared to the observed data (Table 2). The SPACSYS model over-estimated aboveground dry matter accumulation for the Control treatment in the first growing season but slightly under-estimated it for the P added treatment in both seasons (Figure 3). However, the relative errors in final grain yield were ± 15%, with the smallest being only 5.5% for the P added treatment in the 2013–2014 growing season. Furthermore, both simulations and observations showed a difference in dry matter accumulation, especially after GS35 (stem elongation), between the treatments, which suggests that P supply from the soils affected the growth rate. The model slightly over-estimated dry matter accumulation before GS35.

**Figure 4:**
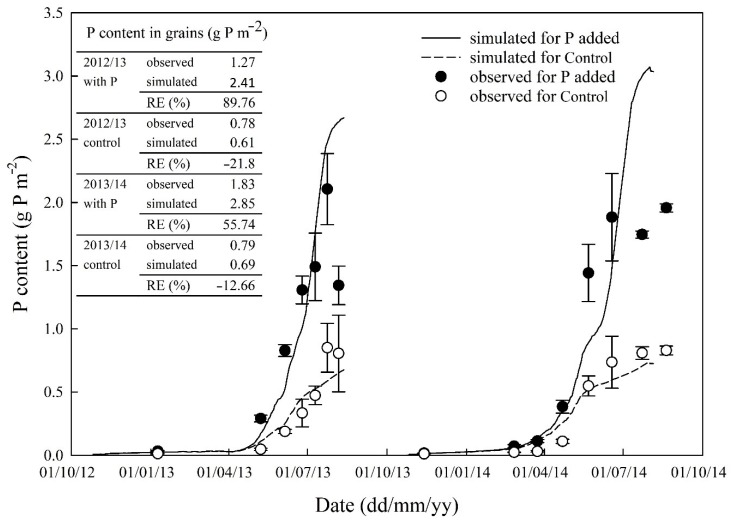
Simulated and observed (three replications) phosphorus content in aboveground dry matter and leaves. The error bars represent the standard deviation.

Simulated P accumulation in aboveground biomass generally followed the observed trend (Table 2) although it showed large discrepancies during the later growing stages. The observed P content in the crop in the P added treatment showed an increase in P accumulation during the later growth stages in the first growing season, but the simulation showed a decrease in rate of accumulation (Figure 4). For the Control treatment, the model over-estimated P uptake before anthesis and under-estimated it after. The phosphorus content in leaves was over-estimated, which might be caused by the defined partitioning coefficients of nutrients absorbed into various organs. 

## 3. Discussions

This study describes the testing of a P module for the SPACSYS model and demonstrates that the SPACSYS model was able to investigate the interactions between C, N, P and water in a single process-based model after the tested P module implemented. The advantage is to investigate not only the dynamics of a single element but also the interactions between them, and in turn, their effects on crop growth using a systems approach. The simulation results show reasonable agreement with observed data in terms of aboveground dry matter accumulation and P content, dynamics of soil water, soil inorganic forms of N and final grain yield under different soil P supply levels under varying climatic conditions. However, large errors between the simulated and observed data occurred when weather was abnormal, e.g., warmer, wetter and more sunshine hours over the 2013–2014 growing season. In the model, however, the negative impact of excess soil water (or waterlogging) on crop growth and partitioning was not considered. It was noted that soil ammonium content was over-estimated in the field of the Control treatment (Figure 2B), which might imply that the soil mineralisation process and the preference of ammonium update by winter wheat were exaggerated under lower soil P concentration. The interaction of soil N and P dynamics under various environmental conditions should be further investigated.

A review on the effects of waterlogging on wheat growth reported that root growth and physiology are adversely affected by soil waterlogging [38]. A field experiment conducted in the UK showed that winter waterlogging affected dry matter accumulation, shoot:root ratio and final yield of cv. Xi-19 [39]. In our study, wheat plants were subjected to saturated conditions during the winter in both growing seasons (Figure 1), which should reduce plant net photosynthesis. Furthermore, plant growth might be subject to the impacts of pests and diseases. However, the model does not consider these. As a result, the model may have over-estimated crop biomass accumulation during the period. During the later stages of the second growing season, the net photosynthesis rate may have been over-estimated because of the establishment of a larger canopy due to favourable weather conditions, which results in enhanced nutrient uptake, accumulated dry matter and P content (Figure 3; Figure 4). There are many processes involved in nutrient cycling further complicated by the interactions between the processes and intricate relationships between these and environmental factors. It is inevitable that the simulations generated from the model created some discrepancies in simulated data compared with observed data. In order to understand and quantify the interaction between nutrients and the impact of nutrient supply on crop growth and partitioning of photosynthate and absorbed nutrients, it might be necessary to design new experiments to monitor nutrient contents in crop and soils under different combinations of N and P supply.

The evidence showed that P plays a fundamental role in controlling resource allocation of plants in response to nutrient enrichment [40]. The model captured the influence of P stress on wheat growth and P content, and final grain yield and P content in grains were reasonably simulated. The simulations suggest that the model estimated the dynamics of P uptake reasonably well (Table 2), which indicates that soil P-cycling was correctly represented in the model. In the observed data, P content in grains rapidly increased at the early stages of grain filling, which is similar to reported observations in rice [41]. Another independent experiment concluded that P deficiency affected harvest index and the root-shoot ratio of barley after anthesis [42]. However, the rate of P remobilised from vegetative tissues to grains was set to a constant in our model. This gave rise to a discrepancy between observed and simulated P contents in grain. As this difference was generated within plants, it could be addressed in further investigations into partitioning of P within the plant and its subsequent remobilisation.

## 4. Materials and Methods

### 4.1. Module of P-cycling

**Figure 5:**
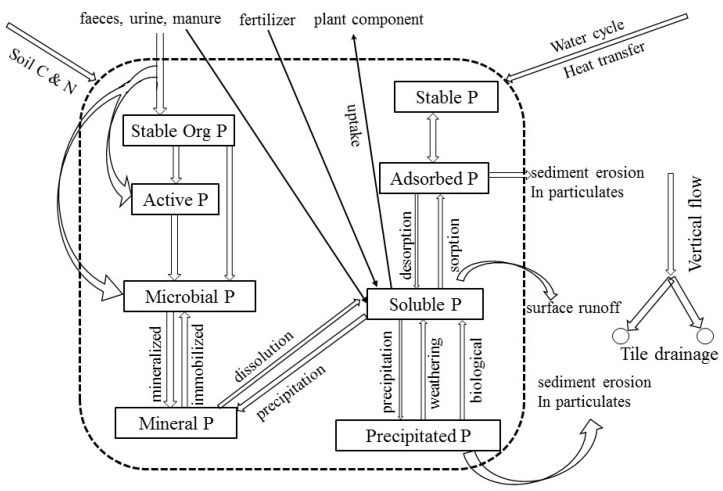
Phosphorus pools (named within the solid rectangle) and their interactions in the P-cycle used in the P module. The arrows between two pools are P flows with specific processes (named next to the arrows) or partitioning (without explanation next to the arrows). The arrows outside the dotted rectangular indicated either the interactions with soil carbon and nitrogen or the influences of soil water redistribution and heat transfer.

The SPACSYS model has been described elsewhere [29,30]. Only the P-cycling module is considered here. In order to match P pools with soil organic C and N pools that were used in the SPACSYS model, soil P was partitioned into soil P pools reflecting those used for soil organic C and N pools as closely as possible. Phosphorus is considered to be present in ups to eight different pools in the SPACSYS model based on its accessibility and extractability, and thus its availability to plants (Figure 5).

Soluble P (Soluble P shown in Figure 5) with main compounds of PO_4_^3−^, HPO_4_^2−^, H_2_PO_4_^−^ is immediately available for uptake by plant roots and can move with soil water in the soil matrix and potentially can leach down the soil profile or be lost to surface waters, typically via subsurface drains. Most of the soluble P taken up by plant roots during a growing season will probably have moved only a few cm or less through the soil to the roots. Inorganic P compounds were divided into three pools apart from the soluble P pool: the stable inorganic P pool (Stable P in Figure 5), the adsorbed P pool and the precipitated P pool, the same as those suggested by Syers et al. [13]. The stable inorganic P pool is strongly bonded and hardly available to plants P. The adsorbed P pool is inorganic phosphate that is attached (or adsorbed) to small particles in the soil and may be released back to the soil solution. The precipitated P pool (or fixed) contains inorganic phosphate compounds that are low in solubility. Phosphate in this pool may remain in soils for years without being made available to plants and may have very little impact on the soil fertility. The inorganic phosphate compounds in the precipitated P pool are more crystalline in their structure and less soluble than those compounds in the stable P pool. In the model, the transformation between inorganic P pools is reversible. Similarly to the inorganic pools, the stable organic and active P pools (Stable Org P and Active P in Figure 5, respectively) are two fractions of organic P. P in microbial biomass (Microbial P shown in Figure 5) is able to connect to the mineral P pool through mineralisation/immobilisation. Mineral P is, in turn, transferred to soluble P via dissolution. The magnitude of the transfer depends on a maximum transfer rate that could be determined with literature or experimental data. The equations and parameters for individual processes in the P module are listed in the Appendix A.

Mass flow, diffusion and root interception are potentially important processes whereby P arrives at the root surface [43]. The uptake of P by plants is an active process that is mediated by specific transport proteins [44]. To reflect the characteristics, the Barber-Cushman model [45,46] that simulates nutrient uptake by roots was incorporated to replace the existing method in SPACSYS for estimating nutrient uptake by plants. As root hairs can play an important role in P uptake [47,48], a modified version of nutrient flux through a cylinder of soil to a root at the centre of the cylinder [49] was adopted:

(1) $$\frac{\partial C_{l}}{\partial t}=\frac{1}{r}\frac{\partial }{\partial r}\left(rD_{e}\frac{\partial C_{l}}{\partial r}+\frac{r_{0}v_{0}C_{l}}{b}-\frac{I_{h}}{b}\right)$$

where *C_l_* is the concentration of nutrient in the soil solution (μmol·cm^−3^), *t* is time (s), *D_e_* is the effective diffusion coefficient for nutrient diffusion through the soil (cm^2^·s^−1^), *r_o_* is the mean root radius (cm), *v_o_* is the mean water influx into the root at the root surface (cm^−1^), *b* is the buffer power of the nutrient adsorbed on the solid phase for the nutrient in solution (-) and *I_h_* is the uptake rate by root hairs per unit volume of root hair zone (μmol·cm^−3^).

The uptake rate of a nutrient at the root is governed by a Michaelis-Menten type equation. Therefore, Equation (1) could be transformed at the root surface (*r* = *r*_0_):

(2) $$\frac{I_{max}\left(C_{l}-C_{min}\right)}{K_{m}+C_{l}-C_{min}}=D_{e}b\frac{\partial C_{l}}{\partial r}+v_{0}C_{l}-I_{h0}$$

(3) $$\frac{I_{max}\left(C_{l}-C_{min}\right)}{K_{m}+C_{l}-C_{min}}=D_{e}b\frac{\partial C_{l}}{\partial r}+v_{0}C_{l}-I_{h0}$$

where *I_max_* is the maximal influx at high concentrations of the nutrient in solution (μmol·cm^−2^·s^−1^), *C_min_* is the concentration below which net nutrient influx ceases (μmol·cm^−3^), *Km* is the nutrient concentration in solution minus *C_min_* where the net influx is one-half *I_max_* (μmol·cm^−3^) and *I_ho_* is the root hair nutrient uptake rate:

(4) $$I_{h0}=\frac{I_{maxh}\left(C_{lh}-C_{min}\right)}{K_{mh}+C_{lh}-C_{min}}$$

where *I_maxh_* is the *I_max_* value for root hairs, *K_mh_* is the *K_m_* value for root hairs and *C_lh_* is the nutrient concentration at the root hair surface.

### 4.2. Linkage with Soil C and N Cycling and Plant Growth

The impact of soil P content on the decomposition rate of organic matter (*D_decom_*, gC·m^−2^·d^−1^) was introduced on top of the existing impact factors in the original version:

(5) $$D_{decom}=k_{max}\cdot D_{pool}\cdot f_{temp}\cdot f_{water}\cdot min\left(f_{CN},f_{CP}\right)$$

where *k_max_* is the specific decomposition rate for a given organic matter pool (d^−1^), *D_pool_* is the biomass of the given organic matter pool (gC·m^−2^), *f_temp_* and *f_water_* are the response functions of the decomposition process to temperature and the soil moisture, respectively; *f_CN_* and *f_CP_* are the unitless response functions to the C:N and C:P ratios of the given organic matter pool, respectively, and were calculated following Jones et al. [50]:

(6) $$f_{CN}=\{\begin{array}{ccccc} 1.0 & & & & \left(CN<25\right)\\ e^{-0.693\left(\frac{CN-25}{25}\right)} & & & & \left(CN\geq 25\right) \end{array}$$

where *CN* is the C:N ratio in the given organic matter pool.

(7) $$f_{CP}=\{\begin{array}{ccccc} 1.0 & & & & \left(CP<200\right)\\ e^{-0.693\left(\frac{CP-200}{200}\right)} & & & & \left(CP\geq 200\right) \end{array}$$

where *CP* is the C:P ratio in the given organic matter pool.

Daily canopy potential net photosynthesis is calculated based on the Hurley Pasture Model (equation 3.2j) [51]. However, the leaf light saturated photosynthetic rate in the equation was modified by the impact of leaf P content:

(8) $$f_{P}=\{\begin{array}{ccc} 0 & & \left(P_{con}\leq P_{min}\right)\\ \frac{P_{con}-P_{min}}{P_{opt}-P_{min}} & & \left(P_{min}<P_{con}<P_{opt}\right)\\ 1 & & \left(P_{con}\geq P_{opt}\right) \end{array}$$

where *P_con_* is the leaf P content (g P·g^−1^ dry matter) that is calculated from simulated P content in leaves and accumulated leaf dry matter (DM), *P_opt_* is the optimal P content (g P·g^−1^ DM) above which the rate is not limited by the leaf P content and *P_min_* is the minimum P content (g P·g^−1^ DM) below which the rate is set to 0 because of the P stress. Both *P_opt_* and *P_min_* are variety specific.

### 4.3. Site Description

To calibrate and validate the model, we used a winter wheat field experiment on the Exhaustion Land plots, which is one of the classical experiments at Rothamsted Research in Harpenden (51°49’N, 0°21’W and 128 m a.s.l.) and situated on Hoosfield and the soil is classified as a Chromic Luvisol (FAO classification) with silty clay loam texture top soil at a 2 m depth [52]. The experiment started in 1852. Since then, the experiment has had several distinct phases in cultivar and fertiliser management [53]. The experimental setup consisted of two treatments: Control (No P was added) and P-added. Each treatment had three replicate plots. The general characteristics of the 0–23 cm depth soils of the six plots (25.6 × 6 m each) are shown in Table 3. From 1986–1992 triple superphosphate was applied 131 kg·ha^−1^ P on the plots for the P-added treatment. Since 1986, all the experimental plots have received annual basal manuring at 300 kg N·ha^−1^ each growing season split into 50, 200 and 50 in March, April and May, respectively, and 124.5 kg·ha^−1^ potassium, and 20 kg·ha^−1^ magnesium sulphate (Mg) every three years (12 kg·ha^−1^ Mg annually since 2009). Since 2000, “maintenance” P has been applied to all the P-added plots. This was applied as 20 kg P·ha^−1^ as triple superphosphate in autumn 2001–2008 and 15 kg P·ha^−1^ 2009–2014. The P-added treatment received triple superphosphate supplying 15 kg P·ha^−1^ incorporated into the soil before wheat was sown in autumn. Winter wheat has been grown on the plots since 1992 [53].

**Figure 6:**
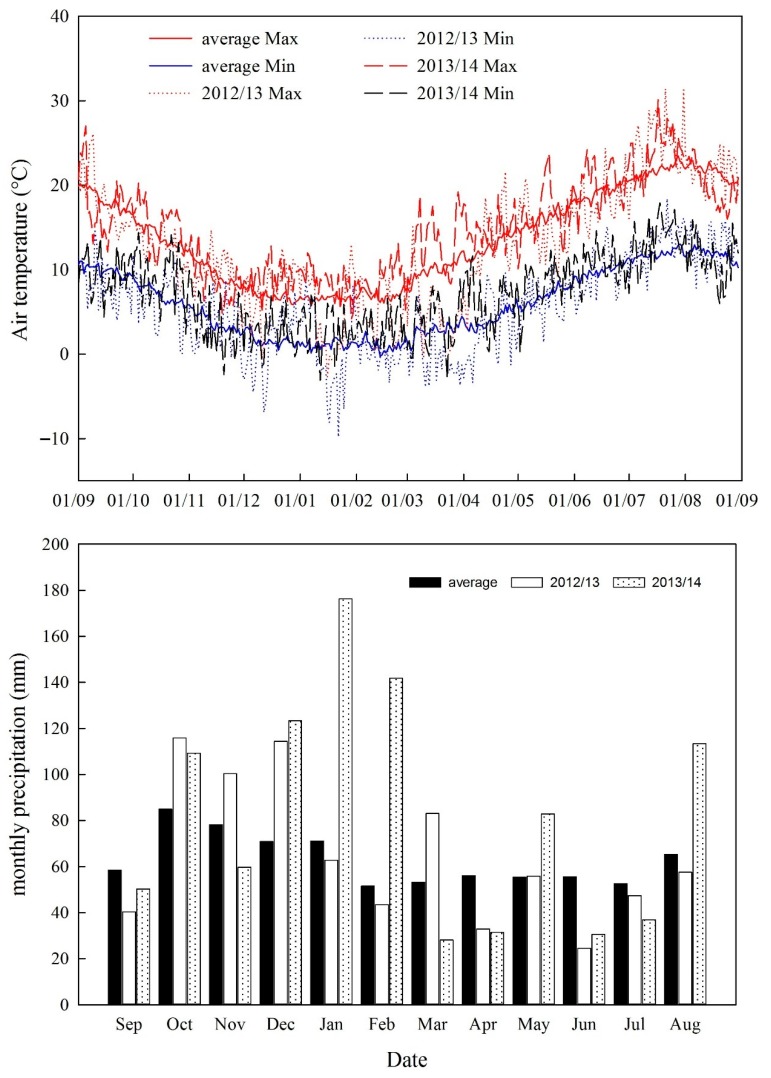
Dynamics of daily maximum and minimum air temperatures and monthly precipitation during the growing seasons (October–July) and historic averages between 1981 and 2010.

Data were collected from the field experiments over two consecutive growing seasons (2012–2013 and 2013–2014). Weather data used for simulations were downloaded from the electronic Rothamsted Archive. Daily maximum and minimum air temperatures and monthly precipitation during the growing seasons were used (Figure 6). The weather conditions contrasted greatly in the two growing seasons. Compared to the mean climatic conditions over the growing season between 1981 and 2010 with a mean temperature of 8.7 °C and a precipitation amount of 611 mm, the first growing season was cooler (7.9 °C) and slightly wetter (672 mm) but the second season was warmer (10.1 °C) and wetter (814 mm). Although the total amount of precipitation over the first growing season was similar to the mean, there was more precipitation during the winter (October–December, 322 mm compared to a mean of 228 mm) of 2012 and it was drier during the vigorous wheat growth period (April–July, 2013, 160 mm compared to a mean of 205 mm). Despite the wetter growing season in 2013–2014, the total sunshine hours (1363 h) were higher than the mean (1243 h) and the first season (1268 h). In general, weather conditions in the second growing season were unusual, whilst in the first season, they could be described as ‘typical’.

### 4.4. Treatments and Measurement

The winter wheat cultivar was Xi-19. Each treatment was replicated in three plots. Soil and plant samples were taken at approximately four weekly intervals throughout the growth period of the wheat, commencing at the Zadoks growth stage (GS) 13 (3 leaves unfolded) [54]. For each sample date, four 0.5 m rows (0.25 m^2^) of wheat on each plot (without repetition) were removed at ground level using secateurs. Soils were sampled using a 2.5 cm diameter stainless steel auger to two depths: 0–23 cm and 23–46 cm. Five cores were taken from each 0.25 m^2^ at each depth and aggregated. 

Harvested wheat samples were washed thoroughly with deionised water to remove any soil contamination. From GS 60 (beginning of anthesis) the wheat plants were separated into stems, leaves and ears and analysed separately. Dry matter was determined by oven drying for 16 h at 80 °C. The dried wheat samples were then ground to pass a 0.5 mm mesh using a Glen Creston Hammer Mill and digested with high purity concentrated nitric and perchloric acids (85/15, *v/v*) in a heating block [55]. Total P concentrations in the plant tissue samples were determined using Inductively Coupled Plasma Optical Emission Spectroscopy (ICP-OES, Perkin Elmer optima 7500 DV, Waltham, MA). 

Soil nitrate and ammonium-N were determined on fresh < 4 mm sieved soil, using 2M potassium chloride as an extractant. Samples were shaken for 2 h and then filtered through a Whatman No.1 filter paper. The supernatant was analysed using an automated colorimetric assay (Skalar SANPLUS System; Skalar, Breda, The Netherlands). The remaining soil was further sieved to <2 mm and dry matter was determined after oven drying for 16 h at 105 °C. Olsen P measurements were determined in extractions from 5 g of air-dried soil with 0.5 M sodium bicarbonate at pH = 8.5 [56]. Soil samples were shaken for 30 min on an orbital shaker (120 rpm, 20 °C), filtered through Whatman 42 filter paper. Phosphorus in the bicarbonate solution was determined by a phospho-molybdenum blue method on the Skalar SAN^PLUS^ System (continuous colourimetric flow analysis).

The measured nutrient concentrations and gravimetric water content of soils were converted into total nutrient contents and volumetric water content using soil bulk density in order to compare them directly with simulated results.

### 4.5. Parameterisation

The parameters used to describe crop growth and development are based on a previous study [33]. Those parameters were used directly in the simulations. SPACSYS was previously parameterised for the processes of soil water, soil heat transformation, C and N cycling [57]. The data collected from the sub-plot of main plot 3 (P added) during the first growing season (2012–2013) on aboveground biomass accumulation and P content in leaves, stems and ears, were used to parameterise crop P uptake, partitioning to leaves, stems and roots and translocation from leaves and stems to grains.

### 4.6. Statistical Analysis

The statistical methods [58] were used to evaluate the performance of the model by comparing simulation results and observed data. Five statistical criteria were included: correlation coefficient (*r*), root mean square error (RMSE), modelling efficiency (EF), relative error (RE) and the coefficient of determination (CD). When a RMSE value is smaller than the RMSE value at the 95% confidence level (RMSE_95%_), this indicates that the simulated values fall within the 95% confidence interval of the measurements. An EF value of 1 means that the simulated values perfectly match the measured values. The closer the model efficiency is to 1, the more accurate the model is. An RE value greater than the RE value at the 95% confidence level indicates that the bias in the simulation is greater than the 95% confidence interval of the measurement. The coefficient of determination (CD) is a measurement of the proportion of the total variance in the observed data that is explained by the simulated data, and was defined by [59]:

(9) $$CD=\frac{\sum_{i=1}^{n}{\left(O_{i}-\bar{O}\right)}^{2}}{\sum_{i=1}^{n}{\left(S_{i}-\bar{O}\right)}^{2}}$$

where *O_i_* are the observed data, *S_i_* are the simulated values, $\bar{O}$ is the mean of the observed data and *n* is the number of samples. CD values can be greater than 1, which indicates that the model describes the measured data better than the mean of the samples [58].

## Figures and Tables

**Table 1:** Model performance statistics on comparison between simulated and observed soil moisture in top (0–23 cm) and sub (23–46 cm) soil layers (*n* = 15) (numbers in parenthesis are the result excluding the unusual sample dated on 25 February 2014).

Statistical Element *	Topsoil Layer		Subsoil Layer	
	P Added	Control	P Added	Control
Correlation coefficient (*r*)	0.86 (0.94)	0.80 (0.89)	0.81 (0.96)	0.67 (0.92)
Modelling efficiency (EF)	0.59 (0.85)	0.52 (0.62)	0.60 (0.89)	0.35 (0.66)
Coefficient of determination (CD)	3.03 (1.22)	2.39 (0.75)	2.08 (0.80)	3.20 (0.63)
Relative error (RE, %)	14.0 (6.67)	13.49 (8.0)	8.24 (2.08)	11.57 (4.48)
RMSE (%)	35.1 (13.8)	29.1 (14.6)	26.3 (9.2)	31.6 (10.9)
RMSE_95%_	35.77 (39.82)	40.55 (44.31)	75.14 (59.03)	51.18 (48.58)

* If all simulated and observed values were the same, then *r* = 1.0; EF = 1; CD = 1; RE = 0; and RMSE = 0.

**Table 2:** Model performance statistics on comparisons between simulated and observed aboveground dry matter and phosphorus content in dry matter (*n* = 14).

Statistical Element *	Aboveground Dry Matter		P Content in Aboveground Biomass	
	P Added	Control	P Added	Control
Correlation coefficient (*r*)	0.99	0.96	0.87	0.82
Modelling efficiency (EF)	0.88	0.91	0.59	0.59
Coefficient of determination (CD)	1.59	1.27	0.63	2.98
Relative error (RE, %)	20.5	−9.0	−1.77	−16.5
RMSE (%)	31.3	31.6	50.4	58.9
RMSE_95%_	117	275	155	300

* If all simulated and observed values were the same, then *r* = 1.0 EF = 1; CD = 1; RE = 0; and RMSE = 0.

**Table 3:** General characteristics of the 0–23 cm soils of the six plots sampled of the Exhaustion Land Experiment.

	Control				P Added			
Main Plot No	3	7	9	Mean (SE)	3	7	9	Mean (SE)
pH (water)	7.2	6.2	7.2	6.82 (0.34)	6.5	6.4	7.1	6.6 (0.21)
Organic C (%)	1.03	0.89	0.86	0.93 (0.05)	1.17	1.04	0.94	1.05 (0.07)
CaCO_3_ (%)	0.55	0.05	0.52	0.37 (0.16)	0.06	0.14	0.42	0.21 (0.11)
Olsen-P (mg kg^−1^)	7.4	6.0	7.4	6.9 (0.47)	31.2	29.0	31.6	30.6 (0.81)
Total-P (mg kg^−1^)	399	351	368	373 (14.15)	596	588	566	584 (8.92)

## Abbreviations

CD	coefficient of determination
EF	modelling efficiency
GS	growth stage
*r*	correlation coefficient
RE	relative error
RMSE	root mean square error

## Appendix A. Equations in the P module

### Appendix A.1. P Mineralisation/Immobilisation

P gross mineralisation is calculated based on C:P ratio in microbial biomass:

(A1) $$M_{P-gross}=P_{microbial}\left(1-\frac{CP_{microbial}}{CP_{critcal}}\right)$$

where *CP_critical_* is critical C:P ratio above which immobilisation occurs, assumed as 28:1 [60], *CP_microbial_* is actual C:P ratio in microbial biomass and *P_microbial_* is P content in microbial biomass.

### Appendix A.2. Dissolution

Dissolution of phosphate minerals occurs when the mineral dissolves and releases P. The algorithm to estimate dissolution from mineral P to soluble P follows Jones et al. [50]:

(A2) $$R_{la}=k_{d}\;\times \left(P_{m}\times \frac{PAI}{1-PAI}-P_{s}\right)\times f_{T}\times f_{w}$$

where *k_d_* is the rate constant for P movement between the two pools (d^−1^) and assumed to be 0.1 under optimum temperature and moisture [61], *P_s_* is soluble P content after fertilisation and incubation, usually for 6 months, *P_m_* is mineral P content, *f_T_* and *f_w_* are response functions of the process to soil temperature and soil moisture, respectively, described in Jones et al. [50], and *PAI* is a P availability index that indicates how much mineral P added to soil remains soluble P on reaching relative equilibrium, and expressed as [50]:

(A3) $$PAI=\frac{P_{s}-P_{s0}}{P_{f}}$$

where *P_so_* is the soluble P content before fertiliser was applied, and *P_f_* is the amount of applied P. The parameter increased with a decrease in CaCO_3_ and clay content, for the calcareous and highly weathered soils, respectively. For slightly weathered soils, it increased with an increase in *P_so_*, base saturation and pH [62].

No net movement between the two pools occurs when they are at equilibrium ($P_{m}=P_{s}\times \frac{1-PAI}{PAI}$). When *P_s_* is higher than its equilibrium concentration, *R_la_* is positive; when *P_s_* is lower, *R_la_* is negative.

### Appendix A.3. Precipitation/Weathering

Phosphate precipitation is a process in which P reacts with another substance to form a solid mineral. Inorganic P precipitates with metal to salts (in acid soil with Al or Fe and Ca in neutral soil). In the current module, only calcium phosphate precipitation was considered. Precipitated inorganic P can be solubilised by bacteria or some plant exudates, named as the weathering process in the module.

Following the ANIMO model [18], precipitation of P takes place immediately when the concentration of the solution exceeds a defined equilibrium buffer concentration (*P_eq_*, g P·m^−3^ soil). This precipitated mineral dissolves immediately when the concentration drops below *P_eq_*. For establishing this equilibrium concentration, ANIMO uses the following relation based on the soil pH:

(A4) $$P_{eq}=0.135\times 10^{-3}\times 3^{5-pH}$$

Calcium phosphate precipitation is a very complex process involving various parameters. It depends on calcium and phosphate ion concentrations, ionic strength, temperature, ion types, pH value and time (solid-solid transformation) [63].

### Appendix A.4. Sorption/Desorption

The phenomenon when soluble P is immobilised into solid forms is defined as sorption in the model. Its reverse process is called desorption. The sorption process assumes that the processes of both absorption and adsorption take place simultaneously. In the model, the two separate processes were not distinguished. Both of them are categorised as sorption, i.e., soluble P being incorporated into a material of a different state and adhering to the surface of another molecule and the molecules being attracted to or attached to solid particles, including the soil. Following the method used in the SWAT model [64], the sorption rate from the soluble P pool to the adsorbed P pool (*P_os_*) was estimated:

(A5) $$R_{sa}=\;\{\begin{array}{c} P_{s}-P_{os}\times \frac{PAI}{1-PAI}\begin{array}{ccccc} & & & & \left(R_{sa}\geq 0\right) \end{array}\\ 0.1\times \left(P_{s}-P_{os}\times \frac{PAI}{1-PAI}\right)\begin{array}{ccccc} & & \left(R_{sa}<0\right) & & \end{array} \end{array}$$

If the value of *R_sa_* is negative, then desorption occurs.

### Appendix A.5. Transfer Between Easily Available P and Fixed P

Following Jones et al. [50], this model simulates slow inorganic P sorption by assuming that easily available mineral P (*P_ia_*, defined as the adsorbed P pool in Figure 5) is in slow equilibrium with a stable mineral P pool (*P_is_*). At equilibrium, *P_is_* is assumed to be four-times *P_ia_*. When not in equilibrium, the rate of movement (*R_as_*) of P between *P_ia_* and *P_is_* is described by

(A6) $$R_{as}=k_{as}\;\times \left(4P_{ia}-P_{is}\right)$$

where *k_as_* (d^−1^) is a rate constant for slow inorganic P adsorption and varies among soils. In calcareous soils, the value ranges from 0.00073 to 0.00079 [65]. In non-calcareous soils, *k_as_* can be calculated from *PAI* [50]:

(A7) $$k_{as}=e^{-1.77PAI-7.05}$$

### Appendix A.6. Soluble P Loss Through Surface Runoff

The primary mechanism of P movement in the soil is by diffusion. Due to the low mobility of soluble P, surface runoff will only partially interact with the soluble P stored in the top 10mm of soil. The amount of soluble P loss (*P_loss_surf_*, g P·m^−2^·d^−1^) is estimated by

(A8) $$P_{loss\_surf}=\frac{R_{\mathrm{runoff}}\cdot P_{con}}{k_{r}}$$

where *R_runoff_* is the surface runoff rate (mm·d^−1^), *P_con_* is soluble P concentration in the top 10 mm soil (g P·mm^−1^ water) and *k_r_* is the P soil partitioning coefficient (cm^3^·g^−1^), i.e., the ratio of the soluble P concentration in the surface 10 mm of soil to the concentration of soluble P in surface runoff.

### Appendix A.7. Phosphorus Attached to Sediment In surface Runoff

Organic and mineral P attached to soil particles may be moved by surface runoff. The amount of P transported with sediment (*P_loss_sed_*, g P·m^−2^) is calculated with a loading function [66]:

(A9) $$P_{loss\_sed}=P_{conc\_sed}*sed*\varepsilon_{p}$$

where *P_conc_sed_* is the concentration of P (the summation of all the P pools except the soluble P pool, g P·g^−1^ sediment) attached to sediment in the top 10 mm, *sed* is the sediment yield on a given day (g), ε_p_ is the P enrichment ratio, defined as the ratio of the concentration of P transported with the sediment to the concentration of P in the soil surface layer and expressed as [67]

(A10) $$\varepsilon_{p}=0.78*{\left(sed_{conc}\right)}^{-0.2468}$$

where *sed_conc_* is the concentration of sediment in surface runoff (g sediment·cm^−3^ water).

### Appendix A.8. Phosphorus Leaching

Because of the low mobility of soluble P, P leaching from the soil profile (gP·m^−2^·d^−1^) is estimated as

(A11) $$P_{loss}=\frac{P_{isconc}}{1000}\cdot q_{d}$$

where P_isconc_ is the concentration of soluble P in a given compartment (gP·m^−3^ water), *q_d_* is ground water flow from the compartment (mm·m^−2^·d^−1^) and the constant of 1000 is for a unit conversion.
